# A methodological framework for real-world performance studies of clinical variant classification platforms at early organizational stages

**DOI:** 10.3389/fgene.2026.1925492

**Published:** 2026-09-14

**Authors:** Vladimir Mitev

**Affiliations:** Helena Bioinformatics, Sofia, Bulgaria

**Keywords:** ACMG/AMP, clinical bioinformatics, inter-laboratory concordance, methodological-disposition taxonomy, multi-source ground truth, rare disease genomics, real-world performance, variant classification

## Abstract

Automated platforms that classify sequence variants under the American College of Medical Genetics and Genomics/Association for Molecular Pathology (ACMG/AMP) framework are increasingly used in rare-disease genomics, yet methodology for characterizing their performance at an early organizational stage is underdeveloped. The two framings most common in the literature are poorly suited to this stage: single-source comparison treats one peer laboratory or curated set as ground truth, which is difficult to defend at evidence depths where qualified laboratories disagree 25%–35% of the time; and formal regulatory adjudication requires multi-adjudicator panels and quality-management infrastructure that small or single-founder developers do not yet have. This paper proposes a methodological framework for Real-World Performance Studies that occupies the space between informal in-house testing and formal regulatory adjudication. The framework has four components: a three-layer performance model that separates analytical, classification, and clinical performance and forces every observation to a locus of attribution; a multi-source ground-truth construction with an explicit, evidence-strength-ordered weighting hierarchy; a six-category methodological-disposition taxonomy that resolves platform-versus-comparator disagreement into characterized categories with distinct action implications, only one of which denotes a classifier defect; and a phased pathway that carries early-stage evidence forward toward eventual regulatory submission. The framework is demonstrated, not validated, on three real-world cohorts (97 scored cases across three classifier versions) of a variant classification platform developed by Helena Bioinformatics. The demonstration illustrates how the method is operationalized. It does not test, validate, or claim superiority for any system. The framework is non-proprietary and is offered for independent adoption and adaptation. Its principal limitations - single-platform demonstration, single peer comparator, a taxonomy developed on the same cohorts that illustrate it, and structurally challenging conflicts of interest - are stated explicitly and are the reason the paper claims a method, not a result. The proposed framework is a conceptual methodology operationalized as a structured human-in-the-loop protocol; it is not a turnkey software package, and empirical use requires version-locked automated outputs plus documented expert review.

## 1 Introduction

### 1.1 The inter-laboratory disagreement landscape

Clinical genomic sequencing is now routine across rare-disease, oncology, prenatal, and population-health settings; whole-genome sequencing reaches a diagnostic yield of approximately 41% in pediatric rare disease (Lionel et al., 2018), and its adoption for first-tier rare-disease diagnosis has been the subject of dedicated implementation guidance (Marshall et al., 2020). This growth has driven demand for automated variant classification under the Richards et al. (2015) framework (Richards et al., 2015). Manual application of the framework’s evidence criteria across hundreds to thousands of variants per case is operationally infeasible at clinical throughput, and the ClinGen Sequence Variant Interpretation (SVI) Working Group has issued a continuous stream of refinements since 2015 - including loss-of-function PVS1 specification (Abou Tayoun et al., 2018), splicing criteria (Walker et al., 2023), computational-predictor calibration (Pejaver et al., 2022), and deprecation of the PP5 reputable-source criterion (Biesecker et al., 2018) - that compound interpretive complexity. Automated platforms encode this evolving framework into systems that produce standardized classification outputs, and those outputs carry clinical weight: variants classified as Pathogenic (P) or Likely Pathogenic (LP) typically trigger management actions, whereas a Variant of Uncertain Significance (VUS) typically defers them.

A persistent feature of this landscape is that qualified laboratories disagree. Inter-laboratory concordance studies under the ACMG/AMP framework have repeatedly documented substantial methodological disagreement - on the order of 25%–35% in five-category classification between qualified laboratories: 66% five-category concordance across nine laboratories in one study (Amendola et al., 2016) and 72%–76% across the same consortium in another (Harrison et al., 2017), with a later analysis across nine genomic-implementation studies reporting lower five-category concordance (54%) but only 11% clinically significant discordance (Amendola et al., 2020). This disagreement is not principally laboratory error. It reflects legitimate methodological evolution: laboratories that apply ClinGen SVI 2020+ specifications - including calibrated computational-predictor thresholds (Pejaver et al., 2022; Bergquist et al., 2025) - can reach systematically different classifications from laboratories applying the historical 2015 base, even when reading the same evidence. The LP-VUS boundary, where the evidence margins separating the two classes are narrow, is a recurring locus of such disagreement (Tavtigian et al., 2018; 2020). Performance characterization methodology must therefore engage this disagreement landscape directly rather than treat peer disagreement as a direct error signal.

### 1.2 Two existing framings and their limits

Two methodological framings dominate platform performance reporting. The first is single-source comparison, in which one peer laboratory’s classifications or one expert-curated set is treated as ground truth. This is appropriate when the reference is an uncontested gold standard at compatible evidence depth - for example, reference materials for variant-detection accuracy. It is difficult to defend for variant classification under ACMG/AMP, where the reference and the platform are independently applying interpretive standards that themselves vary across the comparator population. A platform that disagrees with a single peer laboratory in ∼30% of cases is not necessarily 30% in error; the disagreement may reflect methodological evolution, evidence-access asymmetry, or genuine state-of-the-field uncertainty, and single-source comparison cannot, by construction, distinguish among these.

The second is formal regulatory adjudication, in which multi-adjudicator panels with documented consensus procedures determine binding classifications. This is the correct standard for pivotal regulatory evidence under the *In Vitro* Diagnostic Regulation (IVDR) or equivalent frameworks, but it presupposes substantial infrastructure: paid expert engagement across multiple adjudicators, a mature quality-management system, a full risk-management file, and active conformity-assessment engagement. Such infrastructure is typically unavailable at early organizational stages.

### 1.3 The methodological gap

Between these two framings lies a gap: there is no widely adopted, reproducible methodology for rigorous performance characterization at the organizational stage between informal in-house validation and formal regulatory adjudication. Small-team or single-founder developers building clinical bioinformatics applications - often under partnership arrangements with peer reference laboratories - face a choice between single-source comparison, which is hard to defend at the relevant evidence depth, and formal adjudication infrastructure they do not yet possess. The result, in practice, is either over-claiming (treating peer disagreement as validated accuracy) or under-documenting (deferring rigorous characterization until a later stage and discarding earlier work).

### 1.4 What this paper offers

This paper presents a methodological framework for Real-World Performance Studies designed for that gap: scientifically rigorous, peer-publishable, operationally feasible under early-stage constraints, and forward-compatible with later regulatory work. The framework formalizes four contributions: (1) a three-layer performance model separating analytical, classification, and clinical performance; (2) a multi-source ground-truth construction with an explicit evidence-strength weighting hierarchy; (3) a six-category methodological-disposition taxonomy that resolves platform-versus-comparator disagreement into characterized categories, only one of which denotes a classifier defect; and (4) a phased pathway with foundational-evidence carryforward. The framework also treats conflict-of-interest management as a structural component of the method rather than an afterthought.

Two points define the scope of this paper and should be read as binding throughout. First, the framework is presented as a synthesis and operationalization of existing concepts calibrated for a specific use case, not as unprecedented innovation; its relationship to prior work is set out in the Discussion. Second, the paper is a framework proposal accompanied by a demonstration, not a validation of any platform. The three cohorts described in Section 3 illustrate how the framework operationalizes on a single platform under early-stage constraints; they do not establish the framework’s generalizability or predictive validity, and they make no claim that the demonstrated platform is accurate, best-in-class, or fit for any particular clinical use. Empirical validation of the framework requires independent application by other groups and prospective application to cohorts not used in its development, as discussed in Sections 4.3 and 4.5. So framed, the framework is a testable methodological proposal rather than a settled result: its central claims - that the three-layer model’s attribution discipline correctly separates loci of performance, and that the six-category taxonomy is discriminative under independent blind application - are falsifiable through the conditions specified in Section 2.2 and through prospective, independent multi-platform application as set out in Sections 4.3 and 4.5.

## 2 Methods: the framework

**FIGURE 1:**
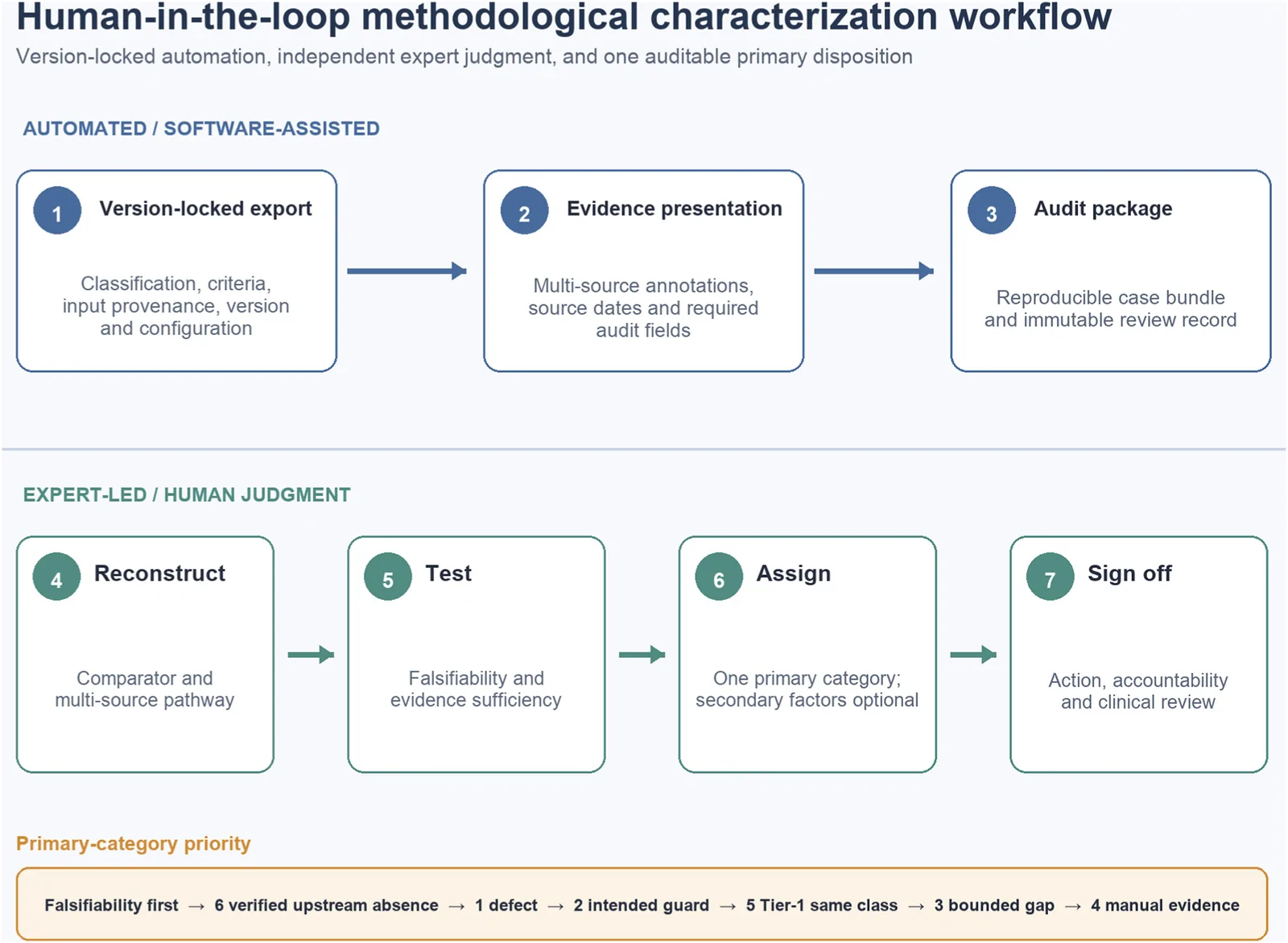
Three-layer performance model. Each performance observation is assigned to its locus of attribution - analytical (Layer 1), classification (Layer 2), or clinical (Layer 3) - preventing the conflated reporting in which an upstream pipeline failure or a clinical-workflow weakness is misattributed to the classification platform.

Operational status of the framework. The framework is a conceptual methodology expressed as an auditable manual protocol with defined software-assisted steps, not a turnkey software workflow. A conforming implementation may automate data ingestion, annotation retrieval, criterion calculation, provenance capture, and report assembly. Human experts remain responsible for judging evidence sufficiency and conflicts, reviewing evidence unavailable from the input VCF, attributing the cause of discordance, selecting the primary methodological disposition, documenting secondary contributing factors, and completing clinical sign-off. The complete human-in-the-loop methodological characterization workflow and primary-category decision priority are shown in Figure 1. Helena Bioinformatics is the demonstrator used in this paper, not a required implementation of the framework.

### 2.1 Three-layer performance model

**FIGURE 2:**
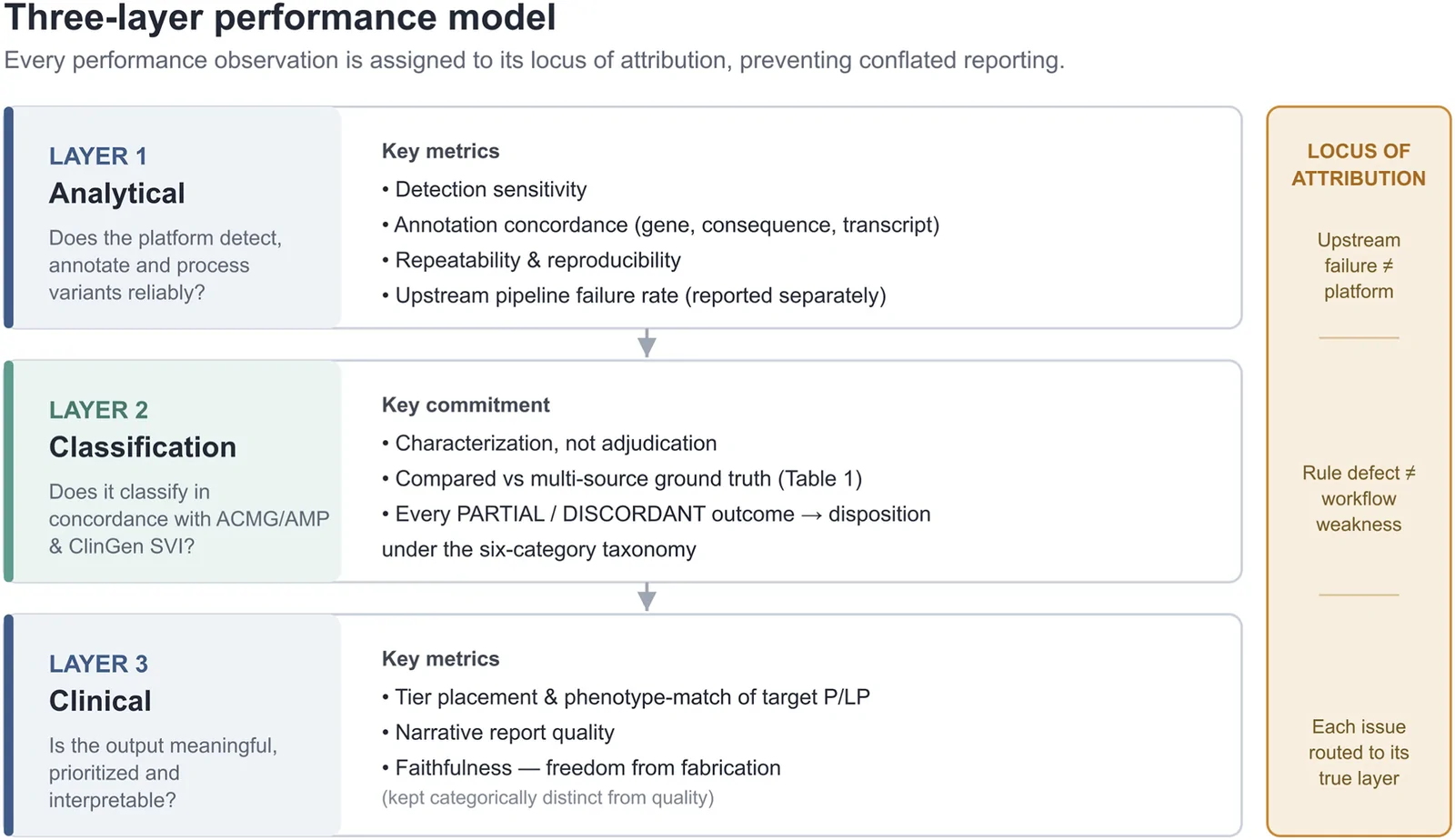
Human-in-the-loop workflow and decision priority. Automated steps produce a version-locked evidence package; expert steps reconstruct the comparator pathway, apply falsifiability conditions, select one primary disposition, record secondary factors, and complete clinical sign-off. The priority rule preserves one analyzable primary outcome while retaining overlap as secondary causal information.

The framework structures performance characterization as three independent and complementary layers, each addressing a distinct aspect of platform behavior. The layered structure is a deliberate methodological choice: it prevents the attribution errors common in conflated performance reporting, where, for example, an upstream sequencing or variant-calling failure is misattributed to the classification platform, or a clinical-workflow weakness is misread as a classification-rule defect. Every performance observation must be assigned to its actual locus of attribution. The three layers and their loci of attribution are summarized in Figure 2.

Layer 1 (Analytical Performance) addresses whether the platform detects, annotates, and processes variants with the technical performance required for diagnostic application. Metrics: detection sensitivity (fraction of variants present in the input VCF that are correctly detected in the classified output); annotation concordance (gene, consequence, transcript against an authoritative reference such as Ensembl or MANE Select); repeatability and reproducibility (identical classification on re-processing and on different infrastructure at the same classifier version); and an upstream pipeline failure rate reported separately and excluded from platform detection sensitivity. The reporting benchmark for diagnostic next-generation-sequencing pipelines is the relevant Clinical and Laboratory Standards Institute guideline (CLSI, 2023), with typical detection and annotation expectations at or above 99%; established standards for validating next-generation-sequencing bioinformatics pipelines apply at this layer (Roy et al., 2018).

Layer 2 (Classification Performance) addresses whether the platform classifies variants in concordance with ACMG/AMP standards, ClinGen SVI specifications, and multi-source reference evidence (Section 2.2). The framework’s central commitment in Layer 2 is mandatory characterization rather than adjudication: every PARTIAL or DISCORDANT outcome against any reference source must be characterized through the protocol of Section 2.5 and assigned a disposition under the taxonomy of Section 2.4. Adjudication in the formal regulatory sense is reserved for later phases (Section 2.7).

Layer 3 (Clinical Performance) addresses whether the platform produces clinically meaningful, prioritized, interpretable output. Metrics include tier placement of target P/LP variants in phenotype-matched output, phenotype-match score against patient phenotype terms, screening rank where applicable, the quality of any platform-generated narrative report, and - treated as a categorically distinct metric - the faithfulness of any narrative report, defined as freedom from factual fabrication (for example, invented disease names, mechanisms, citations, or gene-disease attributions). Faithfulness is kept separate from quality because a report can be low-quality for benign reasons (the target variant was absent from the input and so could not be discussed) without being unfaithful; an unfaithful report, by contrast, asserts something the data do not support, and is a distinct and more serious failure mode.

#### 2.1.1 Clinical translation and professional roles

Layer 3 connects classification output to precision-medicine decisions through phenotype fit, confirmatory testing, multidisciplinary review, and patient preferences; classification alone does not prescribe personalized therapy. Genetic counselors may review evidence, identify discrepancies, communicate residual uncertainty, and coordinate family or segregation testing (Wain et al., 2020). This is not a separate performance layer because professional roles span Layer 2 interpretation and Layer 3 clinical use; their actions are recorded within the relevant layer and the governance log.

### 2.2 Multi-source ground-truth construction

For each variant evaluated, the framework constructs ground truth by triangulation across all available evidence sources with explicit hierarchical weighting by evidence strength, rather than by reference to any single peer laboratory’s clinical classification. The motivation is the empirical disagreement landscape of Section 1.1: because qualified laboratories disagree 25%–35% of the time, a single peer classification cannot serve as definitive truth at these evidence depths. The weighting hierarchy is given in Table 1.

**TABLE 1:** Multi-source reference hierarchy for ground-truth construction.

Source	Evidence type	Weighting
ClinGen Variant Curation Expert Panel (VCEP) curations	Multi-expert consensus (ClinVar 3- or 4-star)	Highest weight; the strongest available anchor where present
Functional studies with clinical follow-up	Published experimental evidence of variant effect plus reported phenotype	High; especially for missense variants in genes with established assays
ClinVar 2-star aggregated submissions	Multiple submitters, criteria provided, no conflicts	Moderate; useful for triangulation
Population genetics	gnomAD allele frequencies and constraint metrics (pLI, observed/expected LoF, LOEUF, missense Z); Kaviar as a provenance-aware novelty resource; regional expression	Moderate; strong for ruling out pathogenicity and supporting LoF intolerance
Calibrated computational predictors	Missense and splice predictors at ClinGen SVI calibrated thresholds (Pejaver et al., 2022; Walker et al., 2023)	Supporting, per calibration
Published case reports and segregation	Peer-reviewed reports in affected individuals or families	Variable, by case quality and segregation strength
Rare-disease and disease-specific knowledge resources	Orphanet/ORDO for disease, phenotype, and gene context; locus-specific and registry databases	Moderate; domain-specific value
Single peer reference laboratory	Single-laboratory clinical classification	Comparator only - not ground truth; informative where it applies different specifications or has access to evidence (segregation, internal series) unavailable to the platform

The peer reference laboratory occupies a comparator-only position by design. This is the commitment that distinguishes the framework from single-source comparison: peer classifications are reported in the context of published inter-laboratory benchmarks, not against a single-source threshold, and every platform-versus-comparator disagreement is characterized and assigned a disposition rather than scored as error.

Multi-source construction does not eliminate genuine evidence uncertainty. Some variants - novel missense in genes without VCEP curation, in uncurated domains, with borderline predictors, and without functional or segregation data - are genuinely at the boundary of available evidence. The framework registers that boundary state explicitly rather than forcing a definitive call.

Reference resources are used according to their evidentiary role rather than treated as interchangeable truth sets. The Association for Clinical Genomic Science (ACGS) rare-disease recommendations supplement ACMG/AMP interpretation in United Kingdom and Ireland practice (Durkie et al., 2024). Kaviar supports provenance-aware assessment of whether a sequence variant has been observed previously, but novelty alone is not evidence of pathogenicity (Glusman et al., 2011). Orphanet and the Orphanet Rare Disease Ontology support standardized disease, phenotype, and gene context, not stand-alone pathogenicity adjudication (Pavan et al., 2017).

#### 2.2.1 Falsifiability conditions

To prevent the methodology from being self-confirming, two conditions are specified in advance under which platform-versus-comparator disagreement is treated as substantive evidence requiring action rather than benign divergence. First, where independent multi-source evidence converges on the comparator’s classification (for example, VCEP curation at 3-star or higher, or two or more independent functional studies with concordant clinical follow-up) and the platform disagrees, the disposition is recorded as evidence-supports-comparator and the case is flagged as a higher-priority remediation candidate, independent of its case-level taxonomy disposition. Second, where the same feature gap recurs across three or more independent cohorts and continues to produce PARTIAL outcomes against comparators at the same rate, the recurring gap is escalated from a documented feature gap (Category 3) to a classifier defect (Category 1). These conditions are forward-looking and constrain future cohorts; they are not applied retroactively to the demonstration cohorts.

### 2.3 Concordance levels

Where multi-source ground truth is feasible, the platform’s classification is compared against it using a structured gradation (Table 2). The framework adopts the FULL/CLINICAL/PARTIAL/DISCORDANT levels established by prior inter-laboratory studies (Amendola et al., 2016; Harrison et al., 2017) and adds NOT EVALUABLE for the setting - absent from those studies - in which the platform processes an input VCF from an external pipeline and the target variant is absent from that input.

**TABLE 2:** Concordance levels.

Level	Definition	Interpretation
FULL	Identical ACMG class (P=P, LP = LP, VUS = VUS, LB = LB, B=B)	Direct agreement; characterization not required
CLINICAL	P-LP boundary, or LB-B boundary	Adjacent classes conventionally treated as clinically equivalent in inter-laboratory concordance reporting
PARTIAL	Adjacent categories differing in management (P/LP-VUS, LB-VUS)	Methodological divergence requiring characterization; not automatic error
DISCORDANT	Opposite-direction (P/LP-B/LB)	Significant divergence requiring detailed investigation
NOT EVALUABLE	Detection failure on the target precludes classification assessment	Reported separately; excluded from concordance fractions; triggers upstream-attribution analysis (Category 6)

NOT EVALUABLE cases are excluded from the concordance denominator on the methodological ground that classification concordance cannot be assessed for a variant the platform never received - consistent with the handling of indeterminate results in diagnostic-accuracy studies. The framework reports both the NOT-EVALUABLE-excluded and the NOT-EVALUABLE-included denominators for transparency.

### 2.4 Six-category methodological-disposition taxonomy

For every Layer 2 outcome that is not FULL concordance with all available references, the framework requires a primary methodological disposition from one of six categories (Table 3). The categories are intentionally action-oriented and may describe co-occurring contributors. To preserve reproducibility, one primary category is selected by the decision priority below and determines the principal action; any additional mechanisms are recorded as secondary contributing factors. This preserves a single analyzable outcome while retaining causal complexity. Only Category 1 denotes a classifier defect.

**TABLE 3:** Six-category methodological-disposition taxonomy.

#	Category	Definition	Action
1	Classifier defect	Output inconsistent with ACMG/AMP 2015 or ClinGen SVI specification	Remediation: change request, retrospective audit, simulation-gated deployment. Only category requiring a fix
2	Validated design behavior	A documented conservative guard firing on its specified trigger; the disagreement is consistent with the guard’s intended design	None; registry records the trigger-configuration validation
3	Feature-gap accumulation at the LP-VUS boundary	Multiple documented, roadmap-tracked scope gaps jointly prevent reaching LP	Roadmap registration with bounded engineering tracking
4	Manual-criteria-dependent automation limitation	Classification depends on manual-curation evidence unavailable from VCF input alone	Long-term automation roadmap only
5	Tier-1 ground-truth concordance	Both reach the same class anchored in Tier-1 ground truth, often via different criteria	None
6	Input-data-layer upstream pipeline failure	Target absent from the input VCF; platform integrity verified separately first	Upstream communication; no platform remediation

Several distinctions are load-bearing. Category 2 (validated design behavior) captures conservative guards that are the intended output - for example, a carrier guard that withholds LP from a single heterozygous loss-of-function variant when the gene’s only established disease mechanism is autosomal-recessive biallelic loss of function (excluding X-linked genes, where a single variant in a hemizygous male can be pathogenic rather than a carrier state), no second qualifying pathogenic variant is observed in trans, and the gene has no documented dominant-acting or gain-of-function mechanism. On that evidence no Likely-Pathogenic combining rule is satisfied; the guard withholds LP as an evidence-level disposition, not as a per-patient claim that the individual is unaffected, and the framework requires genes with dual autosomal-dominant/autosomal-recessive or gain-of-function mechanisms to be excluded from the guard. Because the disposition depends on a fallible mechanism annotation, a disagreement of this kind records a characterized divergence consistent with the guard’s design rather than a proof that the platform is correct. Category 3 (feature gap) and Category 4 (manual-criteria limitation) are both non-defect, but they differ in remediation horizon: Category 3 gaps invite bounded engineering effort (curated-list expansion, criterion implementation), whereas Category 4 limitations reflect the inherent scope of VCF-only automated classification (literature mining, segregation ingestion) and are appropriately deferred. Category 6 (upstream failure) may be assigned only after platform integrity is verified separately - for example, by an end-to-end input-to-database correspondence check across the relevant region - so that an upstream absence cannot mask a platform defect.

#### 2.4.1 Primary-category decision priority

Apply the falsifiability conditions first. Then assign Category 6 only when the target is absent from the received input and platform integrity has been verified; otherwise assign Category 1 for a specification or rule inconsistency; Category 2 for a documented guard operating as designed; Category 5 when platform and comparator reach the same class anchored by Tier-1 evidence despite criterion-level divergence; Category 3 for bounded, implementable scope gaps; and Category 4 when classification depends on evidence that is inherently unavailable to the automated VCF-only workflow. If more than one mechanism remains, the first applicable category in this order is primary and the others are secondary factors. The full decision rules are version-locked in Supplementary Material v1.1.

#### 2.4.2 Worked examples

An incorrect evidence-strength combination is Category 1; a correctly triggered recessive-carrier guard is Category 2; several missing but implementable criteria that jointly prevent an LP call are Category 3; segregation evidence unavailable from the VCF is Category 4; the same final class reached through different criteria and anchored by a VCEP curation is Category 5; and a target absent from the submitted VCF after independent platform-integrity verification is Category 6. A case with both an implementable criterion gap and unavailable segregation evidence is recorded primarily as Category 3, with Category 4 as a secondary factor.

#### 2.4.3 Construct-validity disclosure

The taxonomy was developed iteratively on the demonstration cohorts that subsequently illustrate it; category definitions and action implications were operationalized through interaction with cohort outcomes rather than specified in advance and applied blindly. The distribution of dispositions observed in the demonstration therefore cannot be read as empirical evidence of the taxonomy’s discriminative validity, and the placement of any specific case should not be read as evidence that the same case would receive the same disposition under independent blind application. To enable prospective blind application and independent use, the taxonomy v1.1 specification (definitions, decision rules, action implications) is fixed and provided as version-locked Supplementary Material, to be applied without modification to future cohorts and by independent groups (Data Availability). This limitation is revisited in Sections 4.2, 4.3.

### 2.5 Methodological characterization protocol

For non-FULL outcomes, and for FULL outcomes where criterion-selection differences are informative, the operational sequence is: (1) the platform exports a version-locked classification, applied and withheld criteria, evidence-source provenance, input identifier, software version, and configuration; (2) an expert reviewer reconstructs the comparator and multi-source evidence pathway, including literature, segregation, disease mechanism, and other evidence unavailable from the VCF; (3) the reviewer compares criterion use, evidence strength, and specification versions; (4) the reviewer applies the pre-specified falsifiability conditions; (5) the reviewer assigns one primary disposition using the decision priority in Section 2.4 and records optional secondary contributing factors; (6) the reviewer records the evidentiary basis, required action, responsible role, review date, and taxonomy version; and (7) a qualified expert completes clinical sign-off before any result is used for patient care. Steps 1 and report assembly may be automated; Steps 2–7 require documented human judgment, although software may present evidence and enforce required fields.

#### 2.5.1 Inter-rater reproducibility protocol

Empirical validation should use at least two trained reviewers who independently score the same version-locked cases while blinded to each other’s dispositions and, where feasible, to platform identity. The primary endpoint is the nominal six-category primary disposition. Studies should report raw agreement and Cohen’s kappa with a 95% bootstrap confidence interval; category-specific agreement may be reported descriptively when sparse cells make stable inference difficult. Disagreements are adjudicated only after independent scoring, and both the pre-adjudication and adjudicated records are retained. Training examples must be separate from the validation cohort.

A specific instrument of this protocol is a seven-step upstream-attribution audit for NOT EVALUABLE outcomes, formalized as a reusable triage procedure: (1) independent coordinate validation against an authoritative reference database; (2) end-to-end platform-integrity verification across the relevant region; (3) inspection of the input at the validated target coordinates; (4–6) evaluation of upstream-pipeline attribution candidates (for example, filter-level true-positive loss, or local-assembly failure in complex regions); and (7) reproducibility confirmation by independent re-processing. Only when platform integrity is verified and upstream attribution is established is a Category 6 disposition assigned, with the platform explicitly cleared and the upstream component named with its supporting evidence.

The framework permits and encourages optional academic peer review of completed study reports as supplementary scrutiny, distinct from formal adjudication.

### 2.6 Cohort design and sample-size guidance

Cohort design balances clinical representativeness against sample-size constraints. Cases are drawn from real clinical referrals - not curated for known-answer benchmarking, and consistent with recommendations to validate clinical pipelines by recall testing of real, previously characterized samples (Lavrichenko et al., 2025) - and selected before processing, without performance awareness, for diversity across five axes: clinical domain (at least five distinct domains); inheritance pattern (autosomal dominant, autosomal recessive, X-linked, Y-linked, mitochondrial where applicable); variant type (single-nucleotide variants, indels, splice, structural where in scope); demographic balance where data permit; and source diversity where multiple sources can contribute. Selection preferentially includes variants where multi-source ground truth is achievable. Real-World Performance Studies operate at flexible composition, with the composition rationale documented in advance; the strict positive/negative/edge composition of pivotal cohorts is not applied at this stage. Sample-size guidance is descriptive, not inferential (Table 4).

**TABLE 4:** Illustrative sample-size planning guidance; values are not universal thresholds.

Study type	Illustrative floor	Illustrative target	Rationale
Real-World Performance Study, initial	10	15–20	Descriptive detection of methodology gaps and platform defects; justify against event frequency and population heterogeneity
Real-World Performance Study, expanded	20	25–30	Illustrative descriptive precision only; calculate intervals for the observed event rate and report source-population limits
Methodology Validation Study	hundreds-thousands	thousands+	Inferential claims require estimand-specific power or precision calculations and independent benchmark construction
Pivotal Cohort (future)	30	50+	Illustrative starting point only; pivotal evidence requires outcome-specific power, heterogeneity, and non-evaluable inflation

The numerical values in Table 4 are illustrative planning floors and targets, not universal thresholds. A study should instead justify sample size from its estimand and source population: expected frequency of the event of interest, desired confidence-interval half-width, unit of analysis, non-evaluable inflation, disease and ancestry heterogeneity, recruitment geography, and the feasibility constraints of the rare-disease population. Larger, more heterogeneous or confirmatory studies are required for inferential claims; smaller cohorts remain descriptive and must present uncertainty intervals without implying population-wide precision.

### 2.7 Phased pathway and evidence carryforward

The framework situates Real-World Performance Studies in Phase 1 of an explicitly phased pathway. Phase 1 (Foundation) is the current stage of the developers targeted by the framework. Solo founder or small team without dedicated quality, regulatory, or clinical functions; pre- or seed-stage funding; partnership with one or a few peer reference laboratories; no immediate conformity-assessment engagement. Permitted activities are Real-World Performance Studies, database-derived Methodology Validation Studies, scientific publication, and foundational-evidence accumulation. Deliberately not pursued in Phase 1 are formal external adjudication panels, strict pivotal-cohort composition, multi-site validation, and full quality-management and risk-management documentation. Phase 2 (Growth) introduces formal multi-adjudicator panels, expanded sample sizes, independent operator structures, and multi-site sourcing. Phase 3 (Pre-submission) employs pivotal-cohort evidence for conformity-assessment submission under a mature quality system.

**FIGURE 3:**
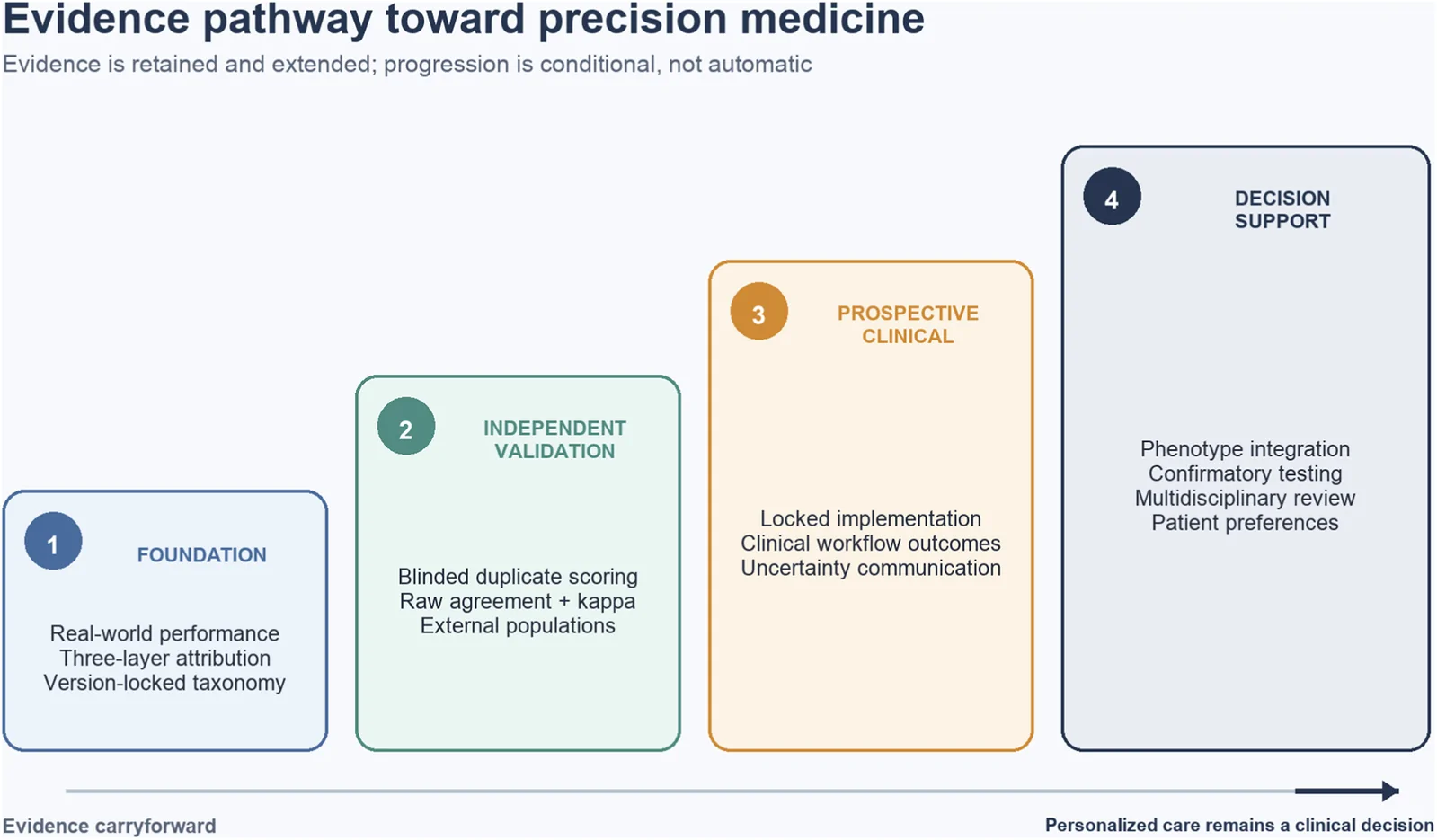
Phased evidence pathway toward precision medicine. Foundation evidence is retained and extended through independent validation, prospective clinical evaluation, and, where appropriate, regulatory or health-system evidence. Progression is conditional rather than mandatory, and classification output reaches personalized care only through phenotype integration, multidisciplinary review, confirmatory testing, and patient preferences.

The defining commitment is foundational-evidence carryforward: Phase 1 performance characterization, identified-defect and remediation history, and published methodology rationale are cited as preliminary evidence in later submission packages rather than discarded. The phased pathway is a permission structure, not a mandatory progression: some platforms may remain at Phase 1 indefinitely, others may stop at Phase 2; the structure makes Phase 3 reachable from Phase 1 evidence without requiring Phase 1 platforms to pre-commit to it. The phased pathway and the carryforward of foundational evidence toward regulatory submission are shown in Figure 3.

### 2.8 Conflict-of-interest management as part of the method

Real-World Performance Studies at early organizational stages frequently operate under structurally challenging conflict-of-interest (COI) configurations: solo-founder operations, advisory relationships with close associates, and partnerships with peer reference laboratories whose personnel may serve in operator or signatory roles. The framework treats COI management as a structural component of the methodology rather than a disclosure afterthought, through a five-category classification (Family, Employment, Professional Affiliation, Financial, Business) aligned with CIOMS and Declaration of Helsinki standards, paired with tailored mitigation rather than avoidance. Mitigations include role exclusion, multi-source triangulation as a structural mitigation against single-source dependency, transparent disclosure, procedural separation, role limitation, and periodic review. This is not incidental to the science: at this organizational stage the most credible defense against the appearance of self-serving evaluation is radical transparency about the conflicts plus a methodology (multi-source ground truth, characterization-not-adjudication) that does not let the developer’s preferred answer become the reference standard. The COI configuration applicable to this paper’s demonstration is disclosed in full in the Conflict of Interest statement.

## 3 Results: framework demonstration

### 3.1 Demonstration design

The framework is demonstrated on three real-world cohorts of a variant classification platform developed by Helena Bioinformatics, comprising 97 scored cases across three classifier versions (Table 5). The platform automates ACMG/AMP 2015 classification with ClinGen-aligned combining rules and conservative guards, processing input VCFs produced by an external whole-genome sequencing provider, in partnership with CellGenetics (Genetic and Medico-Diagnostic Laboratory CellGenetics EOOD; Sofia, Bulgaria), the study’s clinical partner reference laboratory, whose clinical variant classifications serve as the peer comparator throughout this demonstration. The three cohorts illustrate the framework at increasing scale and methodological maturity; they are a demonstration of the method, not a validation of the platform, and no number below should be read as a claim about the platform’s accuracy or fitness for use. The platform is operated on a research-use-only basis under a documented requirement of review by a qualified specialist before any clinical use; it is not CE-marked under the *In Vitro* Diagnostic Regulation (Regulation (EU) 2017/746) and is not registered as an *in vitro* diagnostic medical device.

**TABLE 5:** The three demonstration cohorts.

Characteristic	Cohort 1	Cohort 2	Cohort 3
Role	Retrospective comparator (pre-framework)	First prospective application	Scaled application with structured per-variant re-examination
Classifier version	3.6.4	3.28.0	3.39.1
Scored cases	20	20	57
Clinical domains	11	13	15+
Layer 1 detection	20/20 (100%)	19/20 (95.0%)	184/186 in-scope shared variants (98.9%)
Layer 2 FULL concordance vs. comparator	14/20 cases (70%)	13/19 evaluable cases (68.4%)	102/184 shared variants (55.4%)
FULL + CLINICAL	20/20 (100%)	-	121/184 (65.8%)

Cohort 1 was conducted under a prior single-source paradigm that predates the framework and is reinterpreted here only as a retrospective cross-cohort comparator; it is not a framework application, and its original go/no-go disposition and any clinical-use language are not carried into this paper. Cohorts 2 and 3 are prospective framework applications under pre-specified selection.

The Layer 2 FULL fractions in Table 5 are not directly comparable across cohorts: Cohorts 1 and 2 are scored at the case level (one classification per case), whereas Cohort 3 is scored at the shared-variant level (one comparison per shared variant). The apparent decrease across the row partly reflects this change of unit, together with the differences in classifier version and cohort composition described below, and should not be read as a trend in platform performance.

### 3.2 Cohort 2: first prospective application

Cohort 2 (classifier 3.28.0; 20 cases) was selected before processing under a pre-registered protocol, spanning thirteen clinical domains, eight inheritance patterns, and seven variant types, and including one designated LP control case and four designated compound-heterozygous configurations.

#### 3.2.1 Layer 1

Detection sensitivity was 19/20 (95.0%; Wilson 95% CI 76.4%–99.1%). The single non-detection was resolved by the seven-step upstream-attribution audit to the upstream variant-calling pipeline: independent coordinate validation revealed that the comparator’s reported coordinates were offset by a multi-kilobase distance relative to the authoritative database, an end-to-end correspondence check showed that the platform’s internal pipeline preserved every input variant in the region (equal variant counts at input, after liftover, and as written to the per-session database), and inspection at the validated coordinates confirmed the target variants were never present in the input. Under the audit protocol the absence was therefore attributed to the upstream pipeline rather than to the platform; the most plausible upstream cause was a quality-filter step removing true-positive variants in a complex region. This attribution rests on the objective correspondence check, not on the developer’s judgement.

#### 3.2.2 Layer 2

Against the peer comparator and scored at the case level, 13 of 19 evaluable cases were FULL concordant, six were PARTIAL, none was DISCORDANT, and one was NOT EVALUABLE. FULL concordance was 13/19 evaluable cases (68.4%; Wilson 95% CI 46.0%–84.6%); under the alternative denominator including the NOT EVALUABLE case, 13/20 (65.0%; Wilson 95% CI 43.3%–81.9%). Both fall within the 54%–76% range of five-category FULL concordance reported across multi-laboratory studies (Amendola et al., 2016; Amendola et al., 2020; Harrison et al., 2017), though the confidence intervals at this sample size span a large fraction of the achievable range, and the interpretive content is descriptive consistency with benchmarks rather than precise point estimation. All six PARTIAL cases were LP-VUS adjacent divergences, at the narrow-margin boundary that is a recurring locus of inter-laboratory disagreement (Tavtigian et al., 2018; 2020).

#### 3.2.3 Taxonomy applied

Each non-FULL outcome received a disposition; the taxonomy is illustrated here on representative cases spanning all six categories. A carrier guard and a computational-only LP guard fired on their specified triggers for isolated heterozygous variants in recessive-only genes (Category 2); three jointly accumulating feature gaps at the LP-VUS boundary - a calibrated-versus-internal computational-predictor divergence, an incomplete curated critical-domain list, and an unimplemented supporting criterion - together held a variant below the LP combining threshold (Category 3); a designated LP control case, where the comparator’s LP rested on four manual-curation criteria (case-series, cosegregation, gene-level constraint, and a since-deprecated reputable-source criterion; Biesecker et al., 2018) unavailable from VCF input, was left at VUS by the automated classifier (Category 4); the FULL-concordant cases were anchored in Tier-1 ground truth via VCEP curation, ClinVar 2-star-or-higher aggregates, and clinical guidelines (Category 5); and the upstream pipeline failure above fell outside platform scope (Category 6). One outcome was characterized as a genuine classifier defect (Category 1): a benign-evidence criterion (BP2) specified only for an in-trans observation with a pathogenic variant in a fully penetrant dominant gene (Richards et al., 2015) was being applied to in-trans observations regardless of inheritance pattern, where for a recessive in-trans pair the applicable criterion is instead PM3, which requires a qualifying pathogenic partner variant in trans. Consistent with the framework’s transparency commitment, the defect was reported with full methodological rationale and scheduled for remediation with retrospective audit and simulation-gated deployment; it is reported as a defect in the classifier logic irrespective of whether it altered the resulting classification.

#### 3.2.4 Layer 3

Narrative-report faithfulness was recorded per case; the remaining flagged items were report-quality observations rather than faithfulness failures (an unfaithful report asserts unsupported facts, whereas a low-quality report does not).

### 3.3 Cohort 3: scaled application and structured per-variant re-examination

Cohort 3 (classifier 3.39.1) is the largest and most diverse cohort and the first in which every platform-versus-comparator pathogenicity discordance was re-examined individually under a structured per-variant procedure (described below). Of 60 manifest cases, three were excluded pending comparator reports and one pharmacogenetic-only re-analysis (which classifies no variant in ACMG terms) was segregated, leaving 57 scored cases and 56 cases scoreable at the variant level, with 184 evaluable shared variants. The entire cohort was processed under a single locked classifier version, so the within-cohort comparison is a fixed-classifier comparison.

#### 3.3.1 Layer 1

In-scope detection sensitivity was 184/186 shared variants (98.9%; Wilson 95% CI 96.2%–99.7%). Thirteen NOT EVALUABLE outcomes were separated, by the attribution discipline of the three-layer model, into two methodologically distinct kinds that must not be merged: eleven were out-of-scope structural or copy-number events lying outside the platform’s stated single-nucleotide/short-indel analytic scope, and two were in-scope variant-calling gaps routed to the upstream-attribution audit.

#### 3.3.2 Layer 2

Across the 56 scoreable cases, outcomes were 102 FULL, 19 CLINICAL, 56 PARTIAL, 7 DISCORDANT, and 13 NOT EVALUABLE shared-variant outcomes. FULL concordance was 102/184 (55.4%; Wilson 95% CI 48.2%–62.4%) and FULL + CLINICAL clinical-equivalent agreement was 121/184 (65.8%; Wilson 95% CI 58.6%–72.2%); under the alternative denominator including NOT EVALUABLE outcomes, FULL was 102/197 (51.8%; Wilson 95% CI 44.8%–58.7%). Both the FULL figure (55.4%) and the FULL + CLINICAL figure (65.8%) fall within the 54%–76% range of five-category FULL concordance reported across multi-laboratory studies (Amendola et al., 2016; Amendola et al., 2020; Harrison et al., 2017), with the FULL figure near the lower bound of that range. At 184 evaluable shared variants the Wilson interval is materially tighter than at the 20-case cohorts (roughly ± 7 versus ± 20 percentage points), though the interpretive content remains descriptive. Every one of the 56 PARTIAL components was an LP-VUS or LB-VUS adjacent divergence, and all 7 DISCORDANT outcomes were both-defensible rather than opposite-direction errors.

#### 3.3.3 Structured per-variant re-examination

The 32 platform-versus-comparator pathogenicity discordances were each re-examined end to end: 30 were re-grounded directly from their per-session variant databases (read-only) and the remaining two from the per-case reports, and each was traced through the full classifier path - the loss-of-function (PVS1) eligibility assessment per Abou Tayoun et al. (2018), including established disease mechanism and predicted nonsense-mediated decay; the recessive-carrier guards; the predictor point-sum cap; the computational-only LP guard; the compound-heterozygous partner-validation guard; and the ACMG combining arithmetic - with a disposition recorded per variant only after the full path was read, never from a single criteria string. Under this procedure, none of the 32 discordances was characterized as a Category 1 classifier defect. Each was characterized either to a guard firing on its specified trigger, to a loss-of-function eligibility or combining-rule outcome consistent with the cited specifications, or to the recurring and mechanistically coherent pattern in which the comparator assigned Pathogenic to a single heterozygous variant in a recessive gene while the platform held it at carrier VUS for want of a qualifying second variant in trans. This re-examination was performed by the developer against the platform’s own data, not by an independent or blinded adjudicator; it is therefore a characterization, not an external adjudication, and the absence of a characterized defect in this cohort is provisional - under the framework’s own escalation rule (Section 2.2), a feature gap that recurred at the same rate across three or more cohorts would itself escalate to a defect. The carrier-allele divergences in particular are recorded as methodological divergences whose resolution would require external adjudication, not as evidence that the platform’s classification is correct. Two genuine engineering findings that the procedure surfaced were nonetheless acted on by the developer: a same-codon missense (PM5-type) criterion that had been inert because of a reference-data population gap was rebuilt and re-enabled under the same multi-criterion gating as the other supporting criteria, so that it contributes only in combination and cannot by itself reach a Likely-Pathogenic call; and a compound-heterozygous splice-partner detection gap was widened, with the pathogenicity contribution still gated on a 2-star ClinVar partner so that closing the detection gap could not by itself produce a Likely-Pathogenic call.

#### 3.3.4 Layer 3

Of the 57 cases, 49 produced a narrative interpretation and all 49 were free of gene-disease fabrication; the eight without a narrative reflected a report-coverage gap rather than a faithfulness failure. The remaining Layer 3 observations were prioritization and report-surfacing issues, which the three-layer model assigns to Layer 3 rather than to classification (Layer 2).

### 3.4 Cross-cohort observations

Two designated control variants recurred across cohorts and behaved consistently: a recessive-gene missense control reproduced the same feature-gap (Category 3) disposition, and an autosomal-dominant LP control reproduced the same manual-criteria (Category 4) disposition, in each case classified VUS by the platform against a comparator LP that rested on evidence unavailable from VCF input. A mandatory caveat applies: because the three cohorts were processed under three different classifier versions (3.6.4, 3.28.0, 3.39.1), cross-cohort observations are illustrative and hypothesis-generating, not evidence of variant-level reproducibility under fixed classifier conditions. Distinguishing genuine reproducibility from coincidentally stable output under changed logic, or from a feature-gap pattern surviving multiple versions unremediated, requires prospective application under a single locked classifier version - reserved for future work. The observations are retained as the first systematic cross-cohort linkage produced under the framework, with this limitation explicit.

## 4 Discussion

### 4.1 Relationship to existing approaches

The framework is complementary to, not competitive with, several bodies of prior work. Points-based refinements of ACMG/AMP such as Sherloc (Nykamp et al., 2017; Kobayashi et al., 2025) provide an alternative classification algorithm; the present framework provides performance-characterization methodology applicable regardless of which classification algorithm a platform implements. Comparison-based platform characterizations - for example, single-source benchmarking of prioritization platforms (Manshaei et al., 2022) or cross-tool benchmarking against expert-curated sets (Ghasemnejad et al., 2026) - are methodologically distinct: the former applies the single-source framing whose limits at inter-laboratory evidence depths motivate the multi-source approach here, and the latter operates at cross-tool benchmarking scale rather than single-platform real-world characterization scale. The most conceptually adjacent prior work is the pitfall-taxonomy approach to clinical variant analysis (Coovadia et al., 2023), which categorizes within-platform interpretation errors to strengthen internal quality barriers. The present taxonomy addresses a related but distinct phenomenon at the between-platform level: it characterizes legitimate disagreement between qualified analysts applying defensible methodologies, and it explicitly includes categories where neither party erred (Categories 2 and 5) and categories reflecting tracked limitations rather than specification errors (Categories 3 and 4), with only Category 1 corresponding to the error-categorization framing. The two are complementary for organizations applying both.

### 4.2 What the demonstration does and does not establish

The demonstration establishes that the framework operationalizes end-to-end on real clinical cohorts: every PARTIAL, DISCORDANT, and NOT EVALUABLE outcome across 97 scored cases received a structured disposition. The three-layer model separated an upstream pipeline failure and out-of-scope structural events from platform classification behavior under its attribution discipline; the seven-step audit produced a reusable attribution result; and the structured per-variant re-examination in Cohort 3 produced a per-variant evidentiary trace for each discordance. The inter-laboratory concordance figures observed across cohorts fell within the 54%–76% range of five-category concordance reported across multi-laboratory studies (Amendola et al., 2016; Amendola et al., 2020; Harrison et al., 2017).

The demonstration does not establish that the framework is generalizable, that its taxonomy is discriminative, or that the demonstrated platform is accurate or best-in-class. Three constraints bound the inferential weight of the demonstration in particular. First, the cohorts are small enough that confidence intervals span a substantial fraction of the achievable concordance space; the figures are descriptive, not inferential. Second, the taxonomy was developed on the same cohorts that illustrate it, so the observed disposition distribution is not evidence of discriminative validity. Third, the demonstration is single-platform and single-team. These are not incidental caveats; they are the reason the paper claims a method rather than a result.

### 4.3 Limitations

The following limitations are stated explicitly and are integral to the paper’s claims. (1) Single-platform, single-team demonstration: generalizability requires independent multi-platform application, not performed here. (2) Sample size: the cohorts are appropriate to descriptive Real-World Performance Studies but are not powered for fine-grained inferential claims. (3) Single peer comparator: all cohorts used a single peer reference laboratory; multi-source ground truth mitigates but does not replace multi-laboratory comparison. (4) Conflict of interest: the demonstration was conducted under a structurally challenging COI configuration (solo founder and sole owner; a first-degree relative serving as unpaid scientific advisor; a peer-comparator-employed operator; an external clinical signatory on comparator reports), disclosed in full below; disclosure and structural mitigation do not substitute for the independent multi-party oversight available at later stages. (5) Characterization without external adjudication: Phase 1 employs methodological characterization and, in Cohort 3, an internal, developer-performed per-variant re-examination; neither is an external blinded adjudication, and the dispositions - including the absence of a characterized classifier defect in Cohort 3 - are provisional and await later external review. (6) VCEP coverage: expert-panel curation anchored only a minority of cases, a state-of-the-field limitation. (7) Taxonomy circularity: addressed by fixing the v1.1 specification as version-locked Supplementary Material for application to future cohorts, but the demonstration cohorts themselves are not constrained by that lock. (8) Cross-cohort classifier versioning: cross-cohort observations are illustrative only. (9) Automation provenance: the demonstration cohort reports were produced with substantial automation and AI assistance under human review, disclosed as automation provenance; this is itself a characterization-quality consideration that independent adopters should weigh. (10) Single-population source: all cases derive from one country’s referral population. (11) Phase 1 scope: no pivotal-cohort or regulatory claim is made. (12) Recall on enriched cohorts: the cohorts are positively enriched for known pathogenic variants, so the detection-sensitivity figures are recall on a non-representative variant set and do not estimate population-level sensitivity or specificity. (13) Inter-rater reproducibility: the current single-developer demonstration did not include independent duplicate disposition scoring; consequently, no observed agreement or kappa estimate is claimed. The protocol in Section 2.5 is prospective and requires independent validation.

### 4.4 Generalizability and an invitation to independent adoption

Subject to these limitations, the framework’s design is platform-agnostic in principle: the three-layer model, multi-source ground-truth construction, six-category taxonomy, and phased pathway do not depend on any platform-specific architecture. Principled platform-agnosticism is not a substitute for empirical multi-platform validation, which is why independent adoption is actively invited. The framework is non-proprietary and unpatented; its full specification - the taxonomy v1.1, the three-layer metrics, the weighting hierarchy, and the seven-step audit protocol - is presented here and in the supplement and is free to adopt and adapt (Mitev, 2026). Independent application under independent conflict-of-interest structures would strengthen the framework’s empirical foundation in a way that single-platform development cannot.

### 4.5 Future directions

Anticipated directions include multi-platform validation by independent groups; prospective application under a single locked classifier version to test variant-level reproducibility and the taxonomy under its deposited blind specification; statistical-power studies to refine sample-size guidance per outcome category; engagement with conformity-assessment bodies on the relationship between Phase 1 foundational evidence and formal performance-evaluation requirements; and refinement of the taxonomy as cohort experience accumulates, issued as versioned updates with documented change rationale. Priority should be given to independent, blinded duplicate scoring of the fixed taxonomy with reporting of raw agreement and Cohen’s kappa, followed by external replication across rare-disease populations and clinical settings. Future studies should also evaluate whether the framework’s structured uncertainty communication improves multidisciplinary precision-medicine decisions without overstating classification certainty.

## 5 Conclusion

This paper proposes a methodological framework for Real-World Performance Studies of clinical variant classification platforms at early organizational stages, occupying the space between informal in-house validation and formal regulatory adjudication. The framework formalizes a three-layer performance model, multi-source ground-truth construction, a six-category methodological-disposition taxonomy, and a phased pathway with foundational-evidence carryforward, and it treats conflict-of-interest management as a structural part of the method. A demonstration on three real-world cohorts illustrates how the framework can be operationalized. It is explicitly a proof of concept, not a validation of any platform, and its inferential weight is bounded by single-platform scope, sample size, taxonomy circularity, and disclosed conflicts of interest. The framework is offered as a non-proprietary methodological contribution, and independent adoption under independent conflict-of-interest structures is invited.

## Data Availability

The original contributions presented in the study are included in the article/Supplementary Material, further inquiries can be directed to the corresponding author.
