# Supplementary material for: A methodological framework for real-world performance studies of clinical variant classification platforms at early organizational stages
Source: Front Genet. 2026 Sep 14;17:1925492. doi: 10.3389/fgene.2026.1925492 (PMC13616446; doi:10.3389/fgene.2026.1925492)
Supplement: Supplementary file 1 [file Supplementaryfile1.docx]

**Supplementary Material**

Methodological-Disposition Taxonomy (v1.1): Version-Locked Specification

*A Methodological Framework for Real-World Performance Studies of Clinical Variant Classification Platforms at Early Organizational Stages*

Vladimir Mitev, Helena Bioinformatics, Sofia, Bulgaria

# **1. Purpose and Version Lock**

This document is the version-locked specification of the six-category methodological-disposition taxonomy (v1.1) described in the associated manuscript. It is provided so that the taxonomy can be applied prospectively and blindly, without modification, to cohorts not used in its development and by independent groups working with other platforms.

This specification is issued as version 1.1. It clarifies primary-category priority, permits secondary contributing factors, and adds an inter-rater protocol without changing the six category definitions or their action implications. Any subsequent revision will be issued as a distinct version with a documented change rationale, so that a disposition remains interpretable against the exact rules in force when it was assigned.

Scope of a disposition. A category is assigned to a single platform-versus-comparator disagreement at the level of one variant (or one case, where a study scores at the case level). One primary category is required and determines the principal action. Additional mechanisms may be recorded as secondary contributing factors, but they do not replace or duplicate the primary outcome in analysis. The taxonomy characterizes the methodological structure of a disagreement; it does not render a binding judgment on which classification is clinically correct. Only Category 1 denotes a defect requiring remediation.

# **2. When the Taxonomy Applies**

The taxonomy is applied to every Layer 2 (classification) outcome that is not FULL concordance with all available reference sources. FULL-concordant outcomes require no disposition unless criterion-level differences are being audited. Every non-FULL outcome (CLINICAL, PARTIAL, DISCORDANT, or NOT EVALUABLE, as defined in Section 5) receives exactly one primary category; zero or more secondary contributing factors may also be recorded.

Ground truth for the comparison is constructed by multi-source triangulation with explicit evidence-strength weighting, not by reference to any single peer laboratory. The peer comparator occupies a comparator-only position; a platform-versus-comparator disagreement is characterized and assigned a disposition rather than scored as error.

# **3. Decision Rules**

A non-FULL outcome is resolved in the following order. Evaluate falsifiability first. Then use this primary-category priority: Category 6 after verified upstream absence; Category 1 for a specification or rule inconsistency; Category 2 for a documented guard operating as designed; Category 5 for same-class Tier-1 anchoring despite criterion-level divergence; Category 3 for bounded implementable scope gaps; and Category 4 for evidence inherently unavailable to the automated VCF-only workflow. The first applicable category is primary; additional applicable mechanisms are secondary contributing factors.

**Step 1.** Confirm the outcome is non-FULL against at least one reference source. If FULL with all references, no disposition is required.

**Step 2.** Apply the falsifiability conditions (Section 4). If a condition fires, record its outcome; it may override an otherwise benign disposition or escalate a recurring gap to Category 1.

**Step 3.** If the target variant was absent from the input received by the platform, verify platform integrity independently (Section 6). Only after integrity is confirmed may the outcome be assigned to Category 6.

Step 4. Reconstruct both evidence pathways (platform and reference): which criteria each applied, which were withheld, which evidence was unavailable to the automated workflow, and which specification versions each side used. Apply the primary-category priority stated above and assign the first applicable category.

Step 5. Record the primary disposition, any secondary contributing factors, the supporting evidence, principal action, responsible role, review date, platform and configuration versions, and taxonomy version. Where multi-source evidence supports one side, document the support direction; where it supports both within their respective methodologies, record both-defensible; where genuinely uncertain, record evidence-genuinely-uncertain.

# **4. Falsifiability Conditions (Applied Before Disposition)**

Two conditions are specified in advance under which a disagreement is treated as substantive evidence requiring action rather than benign divergence. They constrain future cohorts and are not applied retroactively to any cohort used to develop the taxonomy.

**Condition 1 (evidence-supports-comparator).** Where independent multi-source evidence converges on the comparator's classification (for example, expert-panel curation at 3-star or higher, or two or more independent functional studies with concordant clinical follow-up) and the platform disagrees, the disposition is recorded as evidence-supports-comparator and the case is flagged as a higher-priority remediation candidate, independent of its case-level category.

**Condition 2 (recurrence escalation).** Where the same feature gap recurs across three or more independent cohorts and continues to produce non-FULL outcomes against comparators at the same rate, the recurring gap is escalated from a documented feature gap (Category 3) to a classifier defect (Category 1).

# **5. The Six Categories**

Each category carries a distinct action implication. One category (1) requires a fix; two (2, 5) require no action; two (3, 4) are roadmap-tracked engineering items; one (6) falls outside platform analytic scope.

| **#** | **Category** | **Definition** | **Action** |
| --- | --- | --- | --- |
| 1 | Classifier defect | Output inconsistent with ACMG/AMP 2015 or ClinGen SVI specification. | Remediation: change request, retrospective audit, simulation-gated deployment. The only category requiring a fix. |
| 2 | Validated design behavior | A documented conservative guard firing on its specified trigger; the disagreement is consistent with the guard's intended design. | None. Registry records the trigger-configuration validation. |
| 3 | Feature-gap accumulation at the LP-VUS boundary | Multiple documented, roadmap-tracked scope gaps jointly prevent reaching Likely Pathogenic. | Roadmap registration with bounded engineering tracking. |
| 4 | Manual-criteria-dependent automation limitation | Classification depends on manual-curation evidence unavailable from VCF input alone. | Long-term automation roadmap only. |
| 5 | Tier-1 ground-truth concordance | Both platform and comparator reach the same class anchored in Tier-1 ground truth, often via different criteria. | None. |
| 6 | Input-data-layer upstream pipeline failure | Target absent from the input VCF; platform integrity verified separately first. | Upstream communication; no platform remediation. |

## **5.1. Load-bearing distinctions**

**Category 2 (validated design behavior).** This captures conservative guards that are the intended output. A representative example is a carrier guard that withholds Likely Pathogenic from a single heterozygous loss-of-function variant when the gene's only established disease mechanism is autosomal-recessive biallelic loss of function; no second qualifying pathogenic variant is observed in trans; and the gene has no documented dominant-acting or gain-of-function mechanism. X-linked genes are excluded, since a single variant in a hemizygous male can be pathogenic rather than a carrier state. On that evidence no Likely-Pathogenic combining rule is satisfied; the guard withholds Likely Pathogenic as an evidence-level disposition, not as a per-patient claim that the individual is unaffected. Genes with dual autosomal-dominant / autosomal-recessive or gain-of-function mechanisms must be excluded from the guard. Because the disposition depends on a fallible mechanism annotation, a disagreement of this kind records a characterized divergence consistent with the guard's design rather than a proof that the platform is correct.

**Category 3 versus Category 4.** Both are non-defect, but they differ in remediation horizon. Category 3 gaps invite bounded engineering effort (curated-list expansion, criterion implementation). Category 4 limitations reflect the inherent scope of VCF-only automated classification (literature mining, segregation ingestion) and are appropriately deferred.

Overlap example. If an implementable missing criterion and unavailable segregation evidence both prevent an LP classification, record Category 3 as primary because it appears first in the decision priority, and record Category 4 as a secondary contributing factor. The primary action is therefore bounded engineering tracking, while the record still preserves the manual-evidence limitation.

**Category 6 (upstream failure).** This may be assigned only after platform integrity is verified separately, for example by an end-to-end input-to-database correspondence check across the relevant region, so that an upstream absence cannot mask a platform defect. The verification procedure is given in Section 6.

# **6. Seven-Step Upstream-Attribution Audit (for NOT EVALUABLE Outcomes)**

Before a NOT EVALUABLE outcome is attributed upstream (Category 6), the following reusable triage procedure is applied. Category 6 is assigned only when platform integrity is verified and upstream attribution is established, with the platform explicitly cleared and the upstream component named with its supporting evidence.

**1.** Independent coordinate validation against an authoritative reference database.

**2.** End-to-end platform-integrity verification across the relevant region (for example, equal variant counts at input, after liftover, and as written to the per-session database).

**3.** Inspection of the input at the validated target coordinates.

**4-6.** Evaluation of upstream-pipeline attribution candidates (for example, filter-level true-positive loss, or local-assembly failure in complex regions).

**7.** Reproducibility confirmation by independent re-processing.

# **7. Concordance Levels (Reference)**

The taxonomy is applied to non-FULL outcomes graded on the following scale. NOT EVALUABLE is excluded from the concordance denominator on the ground that classification concordance cannot be assessed for a variant the platform never received; studies report both the NOT-EVALUABLE-excluded and NOT-EVALUABLE-included denominators.

| **Level** | **Definition** | **Interpretation** |
| --- | --- | --- |
| FULL | Identical five-category classification (P=P, LP=LP, VUS=VUS, LB=LB, B=B). | Direct agreement. No disposition required. |
| CLINICAL | P-LP boundary, or LB-B boundary; same clinical-management implication. | Clinically equivalent. |
| PARTIAL | Adjacent categories differing in clinical management (P/LP-VUS, LB-VUS). | Methodological divergence requiring characterization; not automatic platform error. |
| DISCORDANT | Opposite-direction classifications (P/LP-B/LB). | Significant divergence requiring detailed investigation, regardless of source. |
| NOT EVALUABLE | Target variant absent from the input VCF received by the platform. | Excluded from the concordance denominator; routed through the Section 6 audit. |

# **8. Notes for Independent Application**

This specification is non-proprietary and free to adopt and adapt. It does not depend on any platform-specific architecture: the decision rules, category definitions, and audit procedure apply to any automated classifier compared against multi-source reference evidence.

Groups applying the taxonomy prospectively and blindly are encouraged to record, for each disposition, the criteria each side applied, the specification versions in force, and the supporting multi-source evidence, so that dispositions remain auditable and comparable across cohorts and across groups. The minimum audit record should also include the primary-category decision-rule branch, any secondary factors, reviewer identity, review date, and pre-adjudication status.

**Construct-validity note.** In the associated manuscript, the taxonomy was developed iteratively on the same cohorts that illustrate it; the disposition distribution reported there is therefore not evidence of discriminative validity. Independent, blind, prospective application under this fixed v1.1 specification is the intended path to testing that validity.

Inter-rater protocol. At least two trained reviewers should independently score the same locked cases while blinded to each other's assignments and, where feasible, platform identity. Report raw agreement and Cohen's kappa with a 95% bootstrap confidence interval for the six-category primary disposition. Retain pre-adjudication records; adjudicate only after independent scoring. Use separate cases for training and validation.
